# Choices of (in)action in obesity: Implications for research on treatment and prevention

**DOI:** 10.3389/fpsyt.2022.988495

**Published:** 2022-10-11

**Authors:** Isabel Arend, Michal Schnaider Beeri, Kenneth Yuen

**Affiliations:** ^1^The Joseph Sagol Center for Neuroscience, Sheba Medical Center, Ramat Gan, Israel; ^2^Department of Psychiatry, Icahn School of Medicine at Mount Sinai, New York, NY, United States; ^3^Neuroimaging Center (NIC), Focus Program Translational Neuroscience, Johannes Gutenberg University Medical Center, Mainz, Germany; ^4^Leibniz Institute for Resilience Research, Mainz, Germany

**Keywords:** obesity, risk-taking, passive risk, overweight, inhibition, initiation

## Abstract

The obesity epidemic has crossed social-demographic barriers and is a matter of significant concern. Why do individuals fail to restrain from eating high-calorie foods and fail to follow treatment routines that reduce the risk of health complications? These questions have been addressed through behavioral and brain imaging studies on prefrontal cortex inhibitory mechanisms. Failure to inhibit undesirable behaviors has become a hallmark of obesity. In many life situations, obesity risk is increased by inaction (e.g., not taking blood pressure medication, not following a healthy diet). Risk by inaction has been defined as passive risk-taking, and it is correlated with traits such as procrastination, future time perspective, and cognitive avoidance. To the present, passive tendencies, specifically in the context of risk-taking behaviors, have not been addressed in the obesity literature. We introduce a framework in which active and passive risk-taking behaviors are integrated within the scope of bidirectional models of obesity that describe the brain as both the cause and the consequence of obesity vulnerability. The present perspective aims to foster new research on treatment and prevention, and also on the neurobiology of passive behaviors in obesity and other metabolic conditions.

## Introduction

Obesity as determined by a body mass index (BMI) of 30 kg/m^2^ or higher has become the most prevalent chronic disease in the USA, with 37.9% of adult men and 41.1% of women (1). Obesity can lead to various metabolic complications including type 2 diabetes and cardiovascular conditions that are risk factors for mild cognitive impairment, and dementias (2). Obesity also increases the incidence of psychiatric conditions such as depression and anxiety (3). The impact of obesity in individuals' brain and metabolism can be already identified at young age (4–6).

Psychological processes described within the construct of executive functions have been found to play a significant role in obesity (7). Executive function is a key construct that describes the ability to self-regulate behavior and make decisions that allow the achievement of long-term goals (i.e., being healthy) by overcoming less immediate and more automatic tendencies (6, 8). The prefrontal cortex hosts inhibitory, updating, and shifting mechanisms which have been systematically addressed in obesity (9, 10). Impairment in executive function interferes with processes such as working memory, reasoning, planning, and decision making (8).

Decision-making associated with inhibitory deficits has been consistently reported in obesity (11–14) and shown in individuals without obesity struggling with weight control (15). Individuals with obesity show greater difficulty inhibiting undesirable behaviors and are more sensitive to reward than healthy weight individuals, thereby increasing the probability of engaging in risk-taking behavior (16). Risk-taking behavior in obesity, has traditionally concentrated on the analysis of failures of *inhibition* of undesired behaviors. That is, in the study of actions that increase an individual's risk of gaining weight (e.g., choosing a high caloric dense food; overconsumption of certain foods). There are, however, many situations in which risk is increased by inaction (e.g., not taking the prescribed medication; not engaging in physical activity). This risk-taking tendency has been referred to as passive risk-taking (17, 18), and it constitutes a unique and separate construct from active risk-taking. Furthermore, passive risk choices are perceived as less risky compared to equivalent active risk choices, with less responsibility ascribed to those taking passive compared to active risks (18). The importance of examining passive risk tendencies in the context of obesity is endorsed by studies on self-control, showing that inhibitory behavior fails to predict initiatory behaviors such as the intake of healthy foods (19) which impacts brain and metabolism (20). The present perspective reflects the observation that passive tendencies have been largely neglected in the obesity literature. We propose a framework that integrates active and passive risk-taking within the current bidirectional models of obesity.

### Risk-taking behavior in obesity

The relationship between active and passive risk-taking is central for answering why individuals fail to refrain from eating high-calorie foods and fail to follow treatment routines that *reduce* the risk of health complications. This question is highly intriguing considering that an average adult in industrialized societies has full access to health, nutrition, and exercise information. Nevertheless, the epidemic of obesity is a matter of major concern that has crossed social-demographic barriers (21). In addition, developments in digital medicine have provided consumers with a wide range of digital products that promote healthy practices, such as mobile applications. However, it is important to mention that access to digital products and opportunities for engaging in preventive behaviors such as healthy diet and physical activity are largely constrained by sociodemographic status and other environmental factors. The prevalence of obesity and type 2 diabetes increases at different rates across race/ethnicity groups and sociodemographic conditions among US citizens (22). Therefore, studies that address the brain-behavioral axis underlying obesity and passive risk-taking should be examined while considering potential interactions between the brain, environment (23), and health equity factors (22).

Active risk-taking in obesity has been examined using various tasks (19, for a review). The Iowa Gambling Task (IGT) requires individuals to select cards, with the aim of winning as much money as possible, from four decks, each containing cards that can reward or penalize them using a financial setting (24). The IGT revealed that individuals with obesity relative to individuals without obesity are impaired in decisions under risk compared to decisions under uncertainty (12, 25–28). An increase in risk-taking behavior in obesity was also found using a modified version of the IGT in which the odds for gaining and losing were equal (29), although the effect was observed only for male participants. Similarly, the Balloon Analog Risk Task (BART) was also used to investigate impairments in active risk-taking in individuals with eating disorders and obesity (30). In the BART, participants are asked to pump a balloon to obtain a reward. The balloon may pop at any time during the task at which the money is lost, or participants may press a key to stop to save money.

The Risky-Gains task (RGT) (31) captures similar active risk-taking processes as BART. In the RGT, participants are sequentially shown the numbers 20, 40, and 80, and they are asked to choose the number as a potential reward by a button press. Number 20 is always a safe choice, whereas numbers 40 & 80 are risky options with a certain probability of losing the stated amount. RGT has been used to study individuals with obesity (32, 33). Despite showing a similar proportion of safe vs. risky choices compared to normal weighted controls, individuals with obesity showed differential brain activation patterns in the ventromedial prefrontal cortex and insula during active risk-taking decisions (33).

Schäfer et al. (34) developed the card lottery task (CLT) to examine risk-taking in individuals with severe obesity. The task requires selecting cards from decks with conflicting short- and long-term consequences. This task aimed to resolve a frequent criticism derived from IGT and delay discounting (DDT) in that they do not tackle the conflict between immediate vs. long-term consequences of choices at the same time. Analysis of risk-taking performance of individuals with severe obesity (34), showed that risk-taking performance as measured by CLT was sensitive to changes (i.e., decrease in the number of risky choices) in risk behavior in participants who underwent obesity surgery.

The use of these various active risk-taking tasks shows that, relative to normal-weight individuals, individuals with obesity exhibit decision-making deficits in relation to inhibition, as demonstrated by a higher frequency of risky choices. However, it is still an open question how active risk-taking behaviors relate to initiatory behaviors that lead to the consumption of healthy foods and to engaging in physical activity. Being able or not able to inhibit the urge to consume high-calorie foods does not necessarily translate into healthy eating and exercising.

### Risk-taking by [in]action in obesity

As we mentioned previously, risk can be defined by choices of inaction, for example, not taking prescribed medication or not following a diet to control blood pressure. Passive risk-taking (17) directly relates to deficits in initiating behaviors. Although not addressed under the construct of risk-taking behavior, choices of inaction have been consistently reported in individuals with obesity. Individuals with obesity have been found to have a low propensity to engage in health behaviors such as exercise and the intake of fruits and vegetables (35) and to show low treatment adherence in epidemiological studies addressing cancer treatment (36). Women with obesity have also been found to be less likely to adhere to clinical recommendations for mammography and cervical cancer screening (37). These observations suggest that, as with active risk-taking in obesity, passive risk-taking might go beyond dietary choices and might extrapolate to choices of inaction in various preventive contexts, such as adherence to medicine and conducting health check-ups. As mentioned previously, studies on passive-risk tendencies associated with preventive health practices should be contextualized under health equity issues (22).

Despite its theoretical and practical relevance, empirical research addressing the distinction between inhibitory and initiatory behaviors in obesity is still minimal. The same is true for conditions requiring adherence to diet, physical exercise, and medical recommendations such as metabolic syndrome and type 2 diabetes. The few attempts to distinguish between inhibitory and initiatory mechanisms in obesity were conducted under different theoretical constructs, using various self-report and behavioral measures that challenge the integration of the findings. Using self-report measures, inhibitory behaviors were found to be a successful predictor of the consumption of fat (i.e., inhibitory) and initiatory behaviors of the consumption of fruits and vegetables (i.e., initiatory) in adults (35) and teenagers (38). Inhibition and planning behaviors were measured through a Stroop task and Tower task (39). Planning, which is a facet of goal-oriented behavior, as found to be a reliable predictor of the consumption of fruit and vegetables in adults with obesity (39). Similar tasks such as stop-signal, n-back and operation span tasks along with food consumption self-report measures were used to examine inhibition, planning and updating (19). Inhibitory self-control predicted fat intake, and updating predicted fruit and vegetable consumption.

Studies from health and cognitive social psychology perspectives have provided insights into the importance of initiatory behaviors in various areas, including eating behavior, physical activity, and treatment adherence (35, 40, 41). A trait self-control that describes both inhibitory and initiatory behaviors has been used to predict the engagement in health practices (41). Trait self-control is defined as a stable tendency to adjust to the demands of the environment by inhibiting undesirable behavior and activating goal-beneficial behaviors (40, 42–45). The distinction between these two components of self-control was empirically supported (41). Trait self-control was found to successfully predict health and well-being (46). Recently, the relationship between the initiatory component of self-control was shown to be significantly correlated with passive risk-taking tendencies in the context of risk derived from not taking preventive health measures during the time of COVID-19 (47).

Apart from initiatory behaviors, passive risk-taking is also associated with cognitive tendencies such as self-responsibility, future time perspective and cognitive avoidance (18, 47). Of particular interest among these constructs is future time perspective, which can be experimentally modeled using the delay discounting task (48) and its variants. One of such related computational model examining self-control processes in terms of hyperbolic discount functions describes motivation for impulse control arise from temporally varying outcome values attached to different alternatives, in a process of interpersonal bargaining (49). Intertemporal bargaining centers on the conflict between valuing an option that is presented in a given time just for itself as opposed to valuing an option as an evidence for how you will choose in a bundle of similar future choices (i.e., “I eat this chocolate cake because it is my birthday”). Although these models do not specifically refer to passive risk tendencies, they are potentially relevant for understanding how initiatory behaviors are implemented in various contexts. In a process of recursive interpersonal bargaining, either an immediate behavior or a delayed for reward behavior can prevail simply because one option promises greater discounted reward at the moment of choice.

Our analysis of the literature strongly supports the need to integrate both active and passive tendencies as potential determinants of overweight and obesity. The theoretical advantage of examining inhibitory and initiatory behaviors within the scope of active and passive risk-taking rests on the predictive power of these constructs with respect to behavioral choices. That is, passive risk-taking is highly associated with cognitive tendencies that can be identified, such as self-responsibility, future time perspective and cognitive avoidance (18, 47). For example, individuals who self-report being more future-oriented are more prone to exercise (50–52), more prone to engage in a healthy diet, and to adhere to the prescribed medication for type 2 diabetes and hypertension (53). Therefore, constructs such as future time perspective, might be important mediators for the relationship between passive risk and obesity. In addition, personality dimensions such as conscientiousness and neuroticism associated with specific eating patterns in obesity (51) might also impact active and passive risk-taking tendencies differently.

### Obesity and risk-taking in the human brain

It is well established that decision-making under risk or uncertainty implicates a network of subcortical and cortical areas, including the prefrontal cortex (PFC), limbic regions, and parietal regions (30, 54). For example, a modified version of the BART task was used to examine brain areas associated with risks derived from actively choosing a risky option relative to risks derived from not making a choice (55). Results show that mesolimbic frontal areas, including the dorsomedial prefrontal cortex (dmPFC) and ventromedial prefrontal cortex (vmPFC), are implicated in voluntary but not involuntary risk-taking (55). Using low-frequency repetitive transcranial magnetic stimulation to the right dlPFC during a risk-taking task showed that the disruption of this area leads to risky decisions compared to disruption to the left dlFPC (56).

Only a few studies examine the direct link between risk-taking decisions and obesity (31, 57, 58). Using the Risky Gains task, these studies revealed increasing left inferior frontal/insula activations during risky choices with decreasing BMI and the opposite pattern in the superior midbrain region (48). During risky choices, increased insula activations were correlated with low restrained eating in subjects with obesity (31). Attenuated vmPFC activations were observed in obese subjects (as compared to normal weighted controls) during safe vs. risky choices following a loss (32). These findings suggested potential differences in value representations and interoception between obese and lean people.

The dysregulation of the prefrontal cortex and its role in inhibitory processes in obesity has recently been addressed through studies using non-invasive brain stimulation techniques, for example, transcranial direct current stimulation (tDCS) and transcranial magnetic stimulation (TMS) (59, 60). Although these neurostimulation tools modulate self-control and inhibitory control, their effects on eating behaviors in obese individuals are still inconsistent and difficult to replicate (61–63, for recent reviews). Concerning active and passive risk taking, an interesting direction is suggested by findings showing stimulation to the dlPFC to reduce carbohydrates consumption (61) and to increase physical activity (62), suggesting that stimulation of dlPFC might also impact initiatory behaviors.

To the best of our knowledge currently there exist no published studies directly investigating passive risk-taking and its associated neural correlates. Therefore, we can only examine the neuroimaging correlates of related constructs and indirectly investigate the neural underpinnings of passive risk-taking. As we discussed earlier, one such construct is future time perspective that can be measured by the delay discounting task (63). A recent meta-analysis addressing the brain correlates of delay discounting (64) revealed that the decision to receive immediate gratification, rather than withholding gratification for a better reward, activates a network consisting of the anterior cingulate cortex (ACC), bilateral ventral striatum (VS), left precuneus, right insula and right inferior frontal gyrus (IFG) that processes the intricate interactions between time and values of choices.

Studies using voxel-based morphometry have reported neuroanatomical changes in individuals with obesity in executive function areas, such as medial prefrontal cortex and the temporal pole (61). Recently two large-scale cohort studies (Human Connectome Project & UK Biobank), respectively revealed positive associations between cortical thickness and left superior frontal cortex, left IFG and bilateral parietal cortices (65), and lower subcortical gray matter volume, particularly in ventral and dorsal striatum that associated with obesity (66). In normal-weight participants, individual differences in constructs related to passive risk taking, such as procrastination and future time perspective, were correlated with changes in executive function areas (67). Of main relevance is the finding showing that procrastination and future time perspective overlap in the vmPFC and the parahippocampal gyrus (68). Mediation analysis revealed that the effects of procrastination on gray matter volumes in the parahippocampus and the vmPFC were mediated by future time perspective.

Convergence between neuroimaging correlates of risk-taking decisions and structural changes associated with obesity, particularly the findings in PFC and VS, points to a possible link between the two. Activity in the PFC modulates individual vulnerability to obesity due to its role in executive functions, including decision-making (9). The PFC is connected with cortical and subcortical areas involved in different aspects of eating behavior, such as decision-making, self-regulation, reward sensitivity, and taste evaluation (69). Different roles for the dlPFC and the vmPFC have been identified in response to food-related stimuli (6). While the dlPFC provides inhibitory self-control related to food stimuli, it downregulates taste evaluation within the vmPFC (70). Theories supporting the role of the PFC in obesity suggest that decisions involving healthy food choices depend on the optimum functional coupling of these two subregions (6). That is, optimum control of eating behavior depends on the intact function of the dlPFC to downregulate taste and health evaluation provided by the vmPFC. We propose that choices of inaction, such as those defined within the construct of passive risk-taking, engage the vmPFC to a large extent.

Moreover, in addition to the different roles of the PFC (i.e., ACC, vmPFC, and dlPFC) in food-related choices, there is also growing evidence suggesting that these areas and the hippocampus subserve passive risk-taking-related constructs such as procrastination and evaluation of future consequences (71). The vmPFC is implicated in maximizing gains to prevent future consequences (72, 73). In individuals without obesity, brain imaging studies addressing future time perspectives have reported that the medial PFC, vmPFC, and hippocampus are implicated in future time projections [76-78]. Episodic future thinking during delay discounting tasks reduces the rate of discounting (i.e., reduction of impulsive choices) and increases the functional coupling of the ACC-hippocampus/amygdala (63). This finding is also in agreement with others showing the role of the parahippocampal gyrus in procrastination (71). Coupling between the PFC, including the ACC and hippocampal regions, is also engaged during mental simulations involving future scenarios.

Taken together, these findings are consistent with our proposal that active and passive risk taking partially overlap in the PFC. Although inhibitory processes are consistently found to implicate ACC and dlPFC regions, processes involving evaluation, value attribution, and assessment of future consequences implicate the vmPFC, hippocampus, and parahippocampal area. Future studies should be designed to examine the neuro basis of these two forms of risk and their relation with eating behavior and body composition.

## Discussion

In summary, our analysis of the literature reveals that risk-taking behavior in obesity has mainly been examined through failures to inhibit undesirable behavior. Here we discuss the fact that in real-life situations risk-taking is also derived from choices of inaction. The risks caused by inaction are associated with various psychological traits, such as procrastination, avoidance, and poor initiation. Therefore, it has the potential to play an important role in obesity. We do not suggest that passive risk-taking does not require inhibitory processes. In fact, an important consequence of refraining from taking passive risk is the ability to evaluate the future consequences of a choice and to delay reward (i.e., inhibit). However, successfully preventing passive risk-taking goes beyond being able to inhibit behaviors but to *act* to prevent or to reduce risk. The potential role of inhibition in passive risk behaviors is indeed an important facet of human decision making to be systematically addressed in future research.

We propose to integrate active and passive risk-taking behaviors within the scope of bidirectional models of obesity under which the brain can be both the cause and the consequence of obesity. This bidirectional influence is understood to be mediated by metabolic processes that are triggered by dietary choices and physical activity (54) which impact brain and cognition, specifically executive function (55). Implementing preventive actions that have the potential to impact long-term health depends on individuals' opportunities to implement such actions, for example, sociodemographic and financial factors (23). Therefore, as we mentioned previously, the brain-behavioral axis underlying passive-risk taking choices should be studied while considering potential interactions involving sociodemographic factors and health equity issues (22).

Brain metabolism processes associated with food consumption trigger a cascade of hormone releases, such as insulin, ghrelin, leptin, and glucagon, which in turn impact the brain and cognition (20, 53–55). It has been demonstrated in both human and in mouse models that systemic hyperinsulinemia impairs brain insulin metabolism (20). Behaviorally, the levels of circulating acyl-ghrelin and circulating leptin levels affect decision making, specifically risk preference, suggesting the impact of metabolic states on the brain-behavior axis (58). Executive functions have also been found to be modulated by inflammatory states produced by the immune system response to the presence of stress, pathogens, or tissue injury (48). Inflammation is an important pathophysiological mechanism underlying the effects of nutrition in cognition, as inflammation has been shown to impact brain areas associated with memory, reward, decision making, and affect processing (59–61, 74), all subserving eating habits. Hormonal imbalances and levels of inflammation throughout life might impact brain function by decreasing the ability to resist temptations. Likewise, such disruptions in brain function may increase passive risk-taking choices, making individuals more vulnerable to *not act* to improve their health.

The effects of physical activity and dietary choices on executive function processes well illustrate the bidirectional relationship involving brain and obesity. It has been shown that physical activity increases executive function, promoting inhibitory processes (62). Regular physical exercise (i.e., initiatory behavior) induces inhibitory control and restrains the consumption of unhealthy food (63–65). In the context of physical exercise intervention, transfer effects were observed as physical activity enhanced the consumption of fruits and vegetables, even though the diet was not a target of the intervention (66, 67). Findings showing the effects of exercise on executive function suggest that inhibitory and initiatory factors modulate one another. Less passive risk-taking behavior, such as engagement in physical activity and fruit and vegetable consumption, might impact prefrontal function, reducing active risk-taking behaviors (i.e., succumbing to unhealthy food options). The effects of inhibitory control on brain function were observed following long-term weight loss after laparoscopic sleeve gastrectomy (68). Weight loss significantly decreased food cravings and reduced ghrelin, leptin, and insulin levels (68). Notably, weight loss increased the functional connectivity involving the dorsolateral prefrontal cortex (dlPFC) and pregenual ACC at 1 month and 6 months after the gastrectomy procedure and enhanced cognitive control. This potential bidirectional influence involving active and passive risk choices highlights the importance of tackling both types of risk in preventing obesity. Choices of action that *reduce* risk, such as exercising, also impact cognitive functions that determine the ability to exert inhibitory behaviors that reduce risky behaviors.

Discussing the role of macronutrients and dietary recommendations in obesity is beyond the scope of the present article. However, it is important to mention that the definition of a healthy diet in the context of obesity prevention and treatment goes beyond consuming a certain amount of calories (i.e., caloric intake). Following a healthy diet depends in great extent on initiating (choosing) specific food items rather than simply refraining from overeating. A number of recent studies on nutrition in individuals with obesity and also with type 2 diabetes have shown that not all calories are the same (69). For example, the consumption of different macronutrients such as carbohydrates and fats triggers the different hormonal response (70). In fact, low-carbohydrate diet have a great impact on weight loss and weight control (71, 73). Research addressing active and passive risk taking should benefit from examining the impact initiatory behaviors regarding dietary choices and also physical activity and their associated hormonal response.

Finally, addressing active and passive risk-taking patterns is important for developing digital tools to identify individuals struggling with behavioral adherence and who are at greater risk of progressing to overweight and obesity. Utilizing the latest innovation of assessments using digital biomarkers (75) in combination with machine learning models, could also promote the early detection of active and passive risk patterns. We hope our current perspective will stimulate discussions and further research on active and passive risk-taking in relation to weight control issues, or even expand to other health-related issues. All these research developments will lay the ground for the development of digital health products that promote healthy practices, such as mobile applications.

## Data availability statement

The datasets presented in this article are not readily available because we are submitting a perspective article. Requests to access the datasets should be directed to arend.psy@gmail.com.

## Author contributions

IA, MB, and KY contributed to the conception of the study. IA wrote the first draft of the manuscript. MB and KY wrote sections of the manuscript. All authors contributed to manuscript revision, read, and approved the submitted version.

## Funding

This work was supported by National Institute of Health (USA) awarded MB Grant numbers R01 AG053446, R01 AG051545, and R01AG061093.

## Conflict of interest

The authors declare that the research was conducted in the absence of any commercial or financial relationships that could be construed as a potential conflict of interest.

## Publisher's note

All claims expressed in this article are solely those of the authors and do not necessarily represent those of their affiliated organizations, or those of the publisher, the editors and the reviewers. Any product that may be evaluated in this article, or claim that may be made by its manufacturer, is not guaranteed or endorsed by the publisher.
